# Rhizosphere phosphorus fractions controlled through P fertilization influence wheat infection by *Heterodera avenae*

**DOI:** 10.1186/s12870-025-07399-5

**Published:** 2025-10-03

**Authors:** Mengli Zhao, Pengfei Wang, Xiuli Dong, Siyao Huang, Cihong Wang, Jun Yuan, Wei Qiu, Junhui Chen

**Affiliations:** 1 Key Laboratory of Soil Remediation and Quality Improvement of Zhejiang Province, National Key Laboratory for Development and Utilization of Forest Food Resources, College of Environment and Resources/College of Carbon Neutrality, Zhejiang A&F University, Hangzhou, Zhejiang 311300 China; 2 Key Lab of Organic Based Fertilizers of China, Jiangsu Provincial Key Lab for Solid Organic Waste Utilization, Jiangsu Collaborative Innovation Center of Solid Organic Wastes, Educational Ministry Engineering Center of Resource Saving Fertilizers, Nanjing Agricultural University, Nanjing, Jiangsu 210095 China

**Keywords:** P nutrition, Plant parasitic nematode, Phosphorus availability, Root morphology

## Abstract

**Background:**

*Heterodera avenae *(cereal cyst nematode, CCN) infects wheat and causes severe yield losses. CCN infestations can be decreased by applying phosphate fertilizer in wheat fields, but the underlying mechanisms are still largely unclear.

**Results:**

In this study, the relationships among CCN, wheat root morphological traits, soil P fractions, and soil phosphatase activity in the rhizosphere were investigated with single superphosphate (SSP), monoammonium phosphate (MAP), diammonium phosphate (DAP), and ammonium polyphosphate (APP) application and without phosphate fertilizer (CK) application. APP most effectively inhibited the occurrence of CCN, with the number of cysts decreasing by 48.8%, and was decreased by 31.6%, 33.9%, and 16.4% in SSP, MAP, and DAP treatments, respectively. With APP, the concentrations of Resin-Pi and NaOH-Pi in rhizosphere soil increased significantly, and the proportional lengths of medium (1–3 mm) and coarse roots (> 3 mm) increased, while that of fine roots (0–1 mm diameter) decreased. Moreover, soil phosphatase activity decreased along with highest shoot P accumulation in APP treatment, suggesting that P nutrition provided by APP is more easily absorbed by wheat than the other tested fertilizers. Furthermore, negative relationships were observed between cyst numbers and shoot P accumulation (*r* = -0.87, *p* < 0.001), coarse root length proportion (*r* = -0.59, *p* < 0.01), Resin-Pi (*r* = -0.50, *p* < 0.05), and NaOH-Pi concentrations (*r* = -0.57, *p* < 0.01). SEM analysis indicated that the concentrations of Resin-Pi and NaOH-Pi increased by phosphate fertilizer additions, enhancing shoot P accumulation and inhibiting the occurrence of CCN in wheat.

**Conclusions:**

As an effective *P* source that elevates rhizosphere Resin-Pi and NaOH-Pi, APP not only promotes wheat growth by enhancing P uptake, but also increases the resistance of wheat against CCN. These findings provide an in-depth understanding of the control of CCN through phosphate fertilization management, enabling sustainable agricultural development.

**Supplementary Information:**

The online version contains supplementary material available at 10.1186/s12870-025-07399-5.

## Background

*Heterodera avenae* (cereal cyst nematode, CCN) is a plant parasitic nematode (PPN) widely distributed throughout the world that seriously threatens wheat yield [1, 2]. When wheat plants are infected by CCN, the root absorption of water and nutrients is hindered, resulting in leaf yellowing, plant dwarfing, wilting, and even death [3]. Global wheat yield losses caused by CCN can range from 20% to 70%, resulting in economic losses of about USD $173 billion per year [4, 5]. As most wheat varieties are not CCN resistant, pesticides are widely used [6]. Although nematicides are quickly effective, their use can lead to the repeated occurrence of nematode disease, and frequent use can cause ecological problems, such as environmental pollution [7, 8]. Biological control methods are regarded as environmentally and ecologically friendly, but the lack of biocontrol agent and restrictions due to application conditions makes it difficult to popularize these methods [9, 10]. Although there are many methods for controlling CCN, its occurrence is frequent. One of the main reasons is that intensive agriculture systems that rely on the unsustainable use of mineral fertilizers and pesticides have reduced soil biodiversity and aggravated CCN infestation [2, 11]. Therefore, the implementation of scientifically based fertilization strategies is imperative to control soil-borne pathogens, minimize pesticide dependency, and facilitate sustainable agricultural systems.

As phosphorus (P) participates in diverse biochemical processes, the acquisition of this important, but limited, nutrient affects numerous plant traits as well as crop yields [12]. Applying phosphate fertilizer promotes soil P availability for plants, which contributes to the resistance of plants to many soil-borne diseases, including PPN [13, 14]. Recent research has examined the effects of phosphate fertilizer on PPN. With the increase in superphosphate application, the relative abundance of PPN in farmland has been inhibited by high P concentrations in soil [15]. The relative abundance of PPN in spruce forest soil was reduced by sodium dihydrogen phosphate application [16]. As phosphate fertilizer inhibits the relative abundance of PPN communities in soil, the incidence in crops has also decreased significantly. *Meloidogyne* inhibition rates due to calcium phosphonate, magnesium potassium phosphonate, and potassium phosphonate application were 98%, 66%, and 47%, respectively, in tomato [17]. Siddiqui et al. [18] found that the inhibition rates due to monocalcium phosphate and diammonium phosphate were 30% and 45%, respectively, in tomato. Monoammonium phosphate can effectively reduce the number of CCN cysts in soil, and superphosphate and diammonium phosphate can inhibit their hatching during wheat cultivation [19, 20]. The CCN inhibitory effects differ among phosphate fertilizers, and the mechanism behind them is still unclear.

The soil P supply capacity can be changed through phosphate fertilizer application, thus affecting the absorption and utilization of soil P by plants [21]. Soil P fractions provide a good understanding of effective P and the availability of each P fraction in the soil [22, 23]. The improved Hedley P fraction determination method not only considers the differences between inorganic and organic P, but also comprehensively assesses the dynamic changes in different P fractions in soil [24, 25]. Moreover, the improved Hedley P fractionation method can be adapted to various soil conditions and is being used in an increasing number of studies [26, 27]. Based on this method, soil P is classified as follows: active P (Resin-Pi, NaHCO_3_-Pi, and NaHCO_3_-Po), a fast-cycling P pool, supplying P to plants; moderately active P (NaOH-Pi, NaOH-Po, and Dil.HCl-Pi), a slow cycling P pool, which acts as a potential source of active forms in the short term (days or months) and is a good indicator for the short- to mid-term availability of P; and stable P (Conc.HCl-Pi, Conc.HCl-Po, and Residual-P) remains in the soil for decades without being available for plants [24, 28, 29]. Furthermore, soil P fractions are mediated by soil phosphatase through its control of the transformations between inorganic and organic P pools [12, 23]. Phosphatases are categorized into acid phosphatase (ACP) and alkaline phosphatase (ALP). ACP is excreted by both plant roots and soil microbes, whereas ALP is produced by soil microbes [30]. Phosphate fertilizer application regulates soil P fraction concentrations and phosphatase activity [31]. However, the roles of soil P forms and phosphatase in inhibiting CCN through phosphate fertilizer remains unclear.

Here, we investigated the effects of different types of phosphate fertilizer on (i) cysts, (ii) wheat biological traits, (iii) root morphological traits, (iv) rhizosphere soil P fractions, and (v) soil phosphatase activity. Based on our results, we attempt to clarify the roles of soil P forms, root structure, and soil phosphatase activity on CCN suppression under different phosphate fertilizer applications. Finally, we comment on suitable phosphate fertilizer type to alleviate CCN damage to wheat crops.

## Methods

### Soil preparation and experimental design

The experimental soil was collected from the Institute of Agriculture and Forestry Sciences of Kaifeng in Henan, China (34°76’N and 114°27’E). The soil texture was sandy loam. The chemical properties of the soil were as follows: available N, 58.3 mg kg^−1^; available P, 28.0 mg kg^−1^; available K, 77.7 mg kg^−1^; and pH 8.7. To remove potential confounding influences, the soil used in the experiment was sterilized at 121 °C under high pressure for 2 h to kill CCN. The pre-treated soil was sieved through a 2-mm sieve, and 1 kg of soil was placed in each pot.

The following 5 treatments were set up: no phosphate fertilizer (CK), single superphosphate (SSP), monoammonium phosphate (MAP), diammonium phosphate (DAP), and ammonium polyphosphate (APP). Each treatment was established with five replicates. Except for the CK treatment, pots received 106 mg P kg soil^−1^. Specific amounts of N were added in the form of urea (because MAP, DAP, and APP all contain different amounts of N) to ensure that each pot received 299 mg N kg soil^−1^. According to the local traditional fertilization habits, 199 mg K kg soil^−1^ was applied as potassium chloride in each pot. All fertilizers were thoroughly mixed with the soil per pot alone before planting.

Wheat seeds (*Triticum aestivum* L. cv. Zhoumai 22) were surface-sterilized in 10% v/v H_2_O_2_ for 30 min, rinsed with deionized water, soaked in saturated CaSO_4_ solution for 5 h, then rinsed with deionized water, and germinated in Petri dishes covered with wet filter paper at 22 °C for 3 d. After germination, 6 seedlings were planted in each pot, and 3 seedlings were removed on the 7th day of growth in the greenhouse at 16 °C to retain seedlings with similar growth. CCN (*Heterodera avenae*) inoculation was performed on the 8th day after transplanting into pots, with 500 newly hatched J2 nematodes (incubated at 17 °C for 5 days), and another 500 were applied 2 days later, for a total of 1000 nematodes in each pot. They were cultured in an artificial climate chamber, with 16/8 h of light/dark. The daytime temperature was set at 28 °C, and the nighttime temperature was set at 16 °C. The relative humidity was 65% in the daytime and 75% at night. Soil water content was kept at 70% of field capacity during growth, and pots were watered every 2 days. Samples were collected 6 weeks after inoculation.

### Plant measurements and cyst investigation

The number of tillers was counted, and the height of the wheat plants was measured before sampling. The shoots and roots were sampled and weighed to determine their fresh biomass. Then the seedling strength (stout) was calculated as the following formula:

$$\mathrm{Stout}\;=\;\frac{\mathrm{Shoot}\;\mathrm{fresh}\;\mathrm{weight}}{\mathrm{Height}}$$

The shoot samples were killed at 105 °C for 30 min, dried at 65 °C to a constant weight, and weighed to determine their dry weight. 100 mg of ground, dried samples were used to measure shoot P concentrations according to the procedure of Niederberger et al. [28]. P accumulation in the shoot was calculated for each sample. The fresh root samples were scanned, and the images were analyzed using WinRHIZO software (Regent Instrument, Quebec, Canada) to determine root length in different diameter classes. After that, the root samples were dried and weighted. And the root/top ratio (R/T) was calculated.

All bulk soils were used to determine the number of cysts in each pot. Approximately 980 g of soil was placed in a container, followed by addition of water at a 3:1 water-to-soil ratio (v/w). Then stirred thoroughly for 1 min to release CCN cysts from the soil. Static settlement occurred for 30 s, and the suspension was filtered through a 20-mesh sieve and then an 80-mesh sieve. The whole filtration process was repeated once, and the residue remaining on the 80-mesh sieve was rinsed with a small amount of water for cyst collection. The number of recovered cysts per sample was counted under a stereoscope (Olympus, Tokyo, Japan) and recorded.

### Soil P fraction analysis

To obtain the rhizosphere soils, excess soil was first removed by manually shaking the wheat roots, leaving an approximately 2-mm layer of soil still attached to the roots. Using a sterilized brush, the root-attached soil material was collected as a rhizosphere soil sample [32]. Each rhizosphere soil sample was split into two samples. One sample was air-dried and sieved through a 2-mm mesh and then analyzed for P fractions using sequential fractionation as proposed by Tiessen and Moir [24]. The extractant used for a specific soil P fraction was added to 0.5 g of air-dried soil in the following sequential order: anion exchange resin (Resin-Pi), 0.5 M NaHCO_3_ (NaHCO_3_-Pi and NaHCO_3_-Po), 0.1 M NaOH (NaOH-Pi and NaOH-Po), 1.0 M HCl (Dil.HCl-Pi), concentrated HCl (Conc.HCl-Pi and Conc.HCl-Po), and concentrated H_2_SO_4_–H_2_O_2_ (Residual-P). At each step, the suspension was stirred for 16 h in a shaker (200 rpm) and centrifuged (25,000×g for 10 min at 0 °C), and the supernatant was passed through a 0.45-µm membrane filter and stored prior to colorimetric analysis. Inorganic P was determined following the method described in the Soil Physicochemical Analysis Handbook [33]. The total P concentration in the different extracts (NaHCO_3_-P, NaOH-P, and concentrated HCl-P) was determined using ammonium persulfate digestion. The organic P concentrations were calculated as the difference between total P and inorganic P. The extraction procedure and Hedley P fraction grouping followed that described by Liao et al. [29].

### Soil phosphatase activity investigation

The other rhizosphere soil samples were used to determine the soil phosphatase activity. The activities of ACP and ALP were measured using the fluorometric microplate assay, as described by Saiya-Cork et al. [34]. Briefly, a soil suspension was prepared by homogenizing 1.00 g of fresh soil and 125 mL of tris-base buffer (50 mM, ambient pH) in a 250-mL conical flask for 1 min on a magnetic stir plate. An aliquot of 200-µL soil slurry together with 50-µL fluorometric substrate (200 µM) was added to 96-well flat-black-bottomed microplates (Corning Inc., New York, USA) using an eight-channel pipette (Eppendorf Inc., Hamburg, Germany). The microplates were incubated for 3 h in the dark at 25 °C. The fluorescence was measured using a Tecan Infinite 200 PRO microplate reader (TECAN Group, Ltd., Männedorf, Switzerland) at 360 nm excitation and 450 nm emission.

### Statistical analysis

One-way analysis of variance (ANOVA) was performed using SPSS 21 (IBM SPSS Inc., New York, USA) to assess the significance of treatment effects on cyst number, wheat growth, root morphological traits, shoot P accumulation, rhizosphere P fractions, and phosphatase activity. A heatmap of bivariate Pearson correlations among these indexes was generated using Jamovi 1.6 software. Linear regression analysis and figures were generated using Origin 2024 (OriginLab Inc., Northampton, USA). IBM SPSS Amos 28 (IBM SPSS Inc., New York, USA) was used to construct the structural equation model (SEM). The model fitted by using the maximum-likelihood estimation method. The fitness indexes were χ^2^ = 21.68, df = 18, *p* = 0.329, and RMSEA = 0.041, indicating that the fit of this model met the requirements as described by Shen et al. [35].

## Results

### Soil cysts and plant growth

**Fig. 1:**
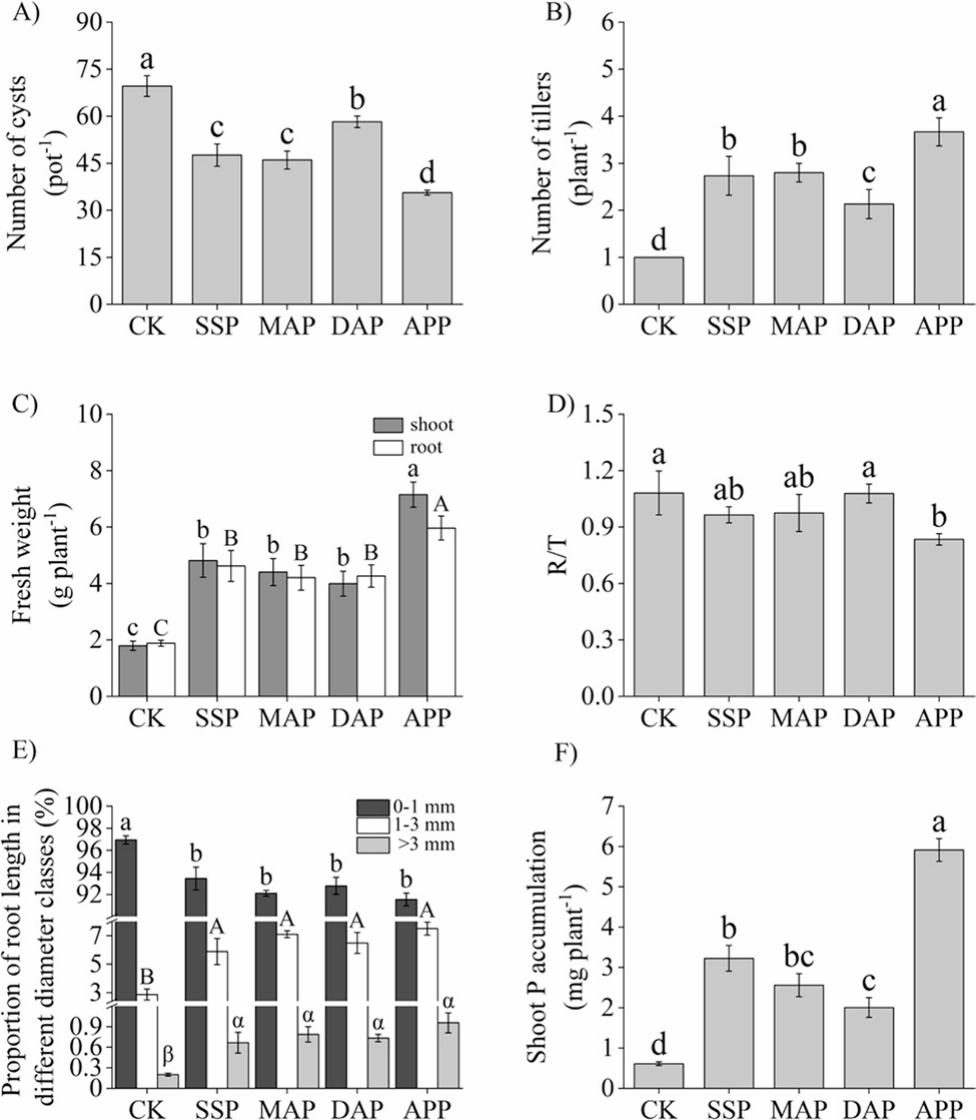
Effects of phosphate fertilizers on the (**A**) number of cysts, (**B**) number of tillers, (**C**) fresh shoot and root weight, (**D**) root-to-shoot ratio, (**E**) proportion of root lengths in different diameter classes, and (**F**) shoot P accumulation. SSP, single superphosphate; MAP, monoammonium phosphate; DAP, diammonium phosphate; APP, ammonium polyphosphate; CK, without phosphate fertilizer. Each value is the mean ± SE of four replicates. Different letters show statistically significant differences among treatments (*p* < 0.05)

After 6 weeks of inoculation with CCN, all four phosphate fertilizer treatments showed disease inhibition on wheat (Fig. 1A). Compared with CK, the APP treatment exhibited the highest inhibition effect, reducing the number of soil cysts by 48.8%. The inhibition effect of the SSP and MAP treatments on cyst number was similar, and inhibition was significantly reduced by 31.6% and 33.9%, respectively, as compared with CK. The inhibitory effect of DAP was the lowest as it reduced cyst number by only 16.4%, as compared with CK.

Wheat tiller number was significantly increased by the four phosphate fertilizer treatments compared with CK, and the promoting effects were in the following order: APP > MAP > SSP > DAP > CK (Fig. 1, Fig. S1). The highest biomass (fresh and dry weights), and seedling strength (stout) and the lowest R/T ratio were observed under APP treatment, but no significant differences were observed among SSP, MAP, and DAP treatments (Fig. 1C and D, Fig. S1C and E). Additionally, soil pH did not vary significantly among the five treatments (Fig. S1B). Root scanning showed that the length ratio of fine roots (0–1 mm diameter, FR) was significantly lower under the four phosphate fertilizer treatments than under CK, while that of medium roots (1–3 mm diameter, MR) and coarse roots (> 3 mm diameter, CR) was significantly higher (Fig. 1E). The total root length and root surface area of plants treated with APP or SSP were significantly higher than those treated with MAP or DAP, and the total root volume was highest in plants treated with APP (Table S1). The P concentration and accumulation in shoots were measured, and the four phosphate fertilizer treatments had significant promoting effects (Fig. 1F, Fig. S1F). They were highest under APP treatment (176.7% and 867.7%, respectively), followed by SSP treatment (94.0% and 427.8%, respectively). APP fertilizer possessed the highest CCN inhibition and wheat growth promotion effects, followed by SSP and MAP, while DAP had the lowest values.

### Rhizosphere soil active P

**Fig. 2:**
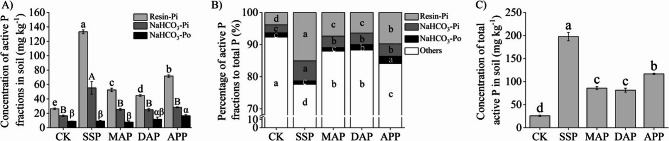
Effects of phosphate fertilizers on soil active P fractions. **A** Concentration of the three active P fractions in the soil. **B** Percentage of active P fractions to total P. **C** Concentration of total active P in soil. SSP, single superphosphate; MAP, monoammonium phosphate; DAP, diammonium phosphate; APP, ammonium polyphosphate; CK, without phosphate fertilizer. Each value is the mean ± SE of four replicates. Different letters show statistically significant differences among treatments (*p* < 0.05)

Resin-Pi concentration in rhizosphere soil was always higher than NaHCO_3_-Pi and NaHCO_3_-Po concentrations, but the concentrations of these active P fractions differed among treatments (Fig. 2). Compared with CK, the Resin-Pi concentration increased significantly under all P treatments, with the highest increase of 407.4% measured under the SSP treatment, followed by an increase of 173.4% under the APP treatment. The NaHCO_3_-Pi concentration was significantly higher in rhizosphere treated with SSP than all other treatments. The NaHCO_3_-Po concentration was not different between the APP and DAP treatments, but the NaHCO_3_-Po concentration was significantly higher under APP treatment than under the CK, SSP, and MAP treatments (Fig. 2A).

Comparing the differences in the percentages of the three active P fractions in total P among treatments showed that the proportions of Resin-Pi and NaHCO_3_-Pi under SSP treatment (15.1% and 6.3%, respectively) were much higher than those in other treatments (Fig. 2B). The trend in the proportion of Resin-Pi showed the following order among treatments: SSP > APP > MAP ≈ DAP > CK. The proportion of NaHCO_3_-Po was highest under APP treatment, accounting for 2.3%.

Compared with CK, the total active P concentration in rhizosphere was significantly increased in all four P treatments (Fig. 2C). Among them, total active P concentration was highest under SSP treatment, followed by APP treatment, but there were no significant differences between MAP and DAP treatments.

### Rhizosphere soil moderately active P

**Fig. 3:**
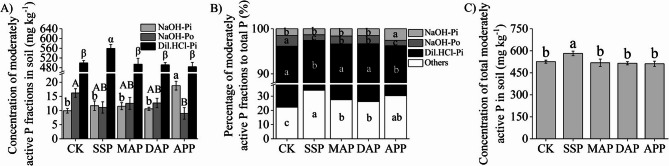
Effects of phosphate fertilizers on soil moderately active P fractions. **A** Concentration of the three moderately active P fractions in the soil. **B** Percentage of moderately active P fractions to total P. **C** Concentration of total moderately active P in soil. SSP, single superphosphate; MAP, monoammonium phosphate; DAP, diammonium phosphate; APP, ammonium polyphosphate; CK, without phosphate fertilizer. Each value is the mean ± SE of four replicates. Different letters show statistically significant differences among treatments (*p* < 0.05)

For the moderately active P fractions in rhizosphere, Dil.HCl-Pi concentration was significantly higher than NaOH-Pi and NaOH-Po concentrations in all P treatments (Fig. 3). As shown in Fig. 3A, the Dil. HCl-Pi concentration in the SSP treatment was significantly higher than that in the other treatments, while there were no significant differences among the other treatments. Compared with CK, the NaOH-Pi concentration was significantly increased under APP treatment, while the NaOH-Po concentration was significantly decreased. There were no significant differences in the NaOH-Pi and NaOH-Po concentrations among the SSP, MAP, and DAP treatments.

Comparing the proportions of the three moderately active P fractions in total P among treatments showed that the proportion of Dil.HCl-Pi in the SSP and APP treatments (63.3% and 65.9%, respectively) were lower than that in the CK (Fig. 3B). The proportion of NaOH-Pi was highest in the APP treatment (2.6%). The ratio of NaOH-Po in the four P treatments was lower than that of CK. Comparing the total moderately active P concentration among treatments showed that it was significantly higher in the SSP treatment than in the other treatments (Fig. 3C). In addition, no significant differences were observed between MAP, DAP, and APP treatments.

### Rhizosphere soil stable P

**Fig. 4:**
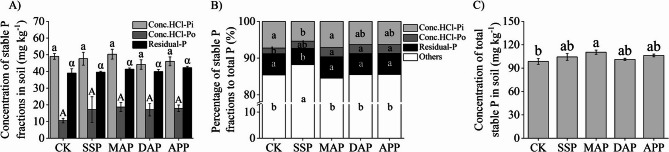
Effects of phosphate fertilizers on soil stable P fractions. **A** Concentration of the three stable P fractions in the soil. **B** Percentage of stable P fractions to total P. **C** Concentration of total stable P in soil. SSP, single superphosphate; MAP, monoammonium phosphate; DAP, diammonium phosphate; APP, ammonium polyphosphate; CK, without phosphate fertilizer. Each value is the mean ± SE of four replicates. Different letters show statistically significant differences among treatments (*p* < 0.05)

Compared with CK, there were no significant differences in the Conc.HCl-Pi, Conc.HCl-Po, and Residual-P concentrations under different phosphate fertilizer treatments (Fig. 4A). However, the proportions of Conc.HCl-Pi and Residual-P in total P under SSP treatment (5.4% and 4.5%, respectively) were lower than that of CK (Fig. 4B). The proportion of Conc.HCl-Po under MAP, DAP, and APP treatments (2.6%, 2.5%, and 2.4%, respectively) were higher than that in CK. Comparing the differences in the total stable P concentration among treatments showed that it was significantly higher under MAP treatment than under CK, but there were no significant differences between the other treatments (Fig. 4C).

### Rhizosphere phosphatase activity

**Fig. 5:**
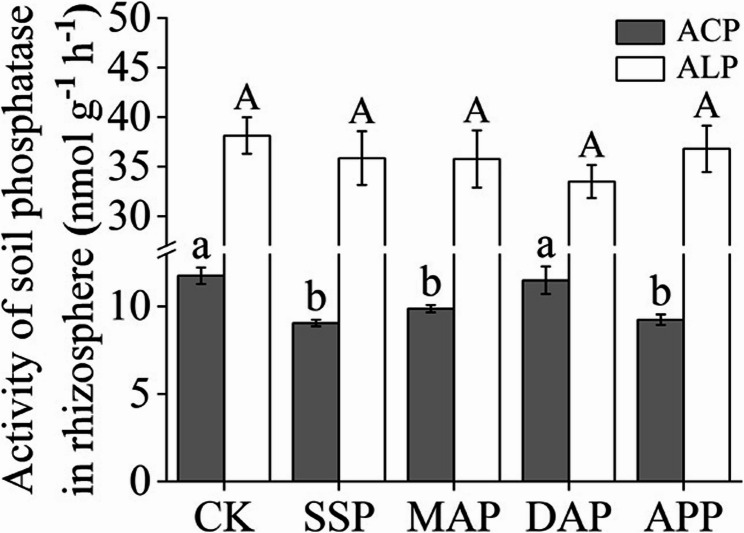
Effects of phosphate fertilizers on soil phosphatase activity. SSP, single superphosphate; MAP, monoammonium phosphate; DAP, diammonium phosphate; APP, ammonium polyphosphate; CK, without phosphate fertilizer. Each value is the mean ± SE of four replicates. Different letters show statistically significant differences among treatments (*p* < 0.05)

Phosphatase activity was measured in wheat rhizosphere soil, and the results are shown in Fig. 5. The activity of ACP did not differ among SSP, APP, and MAP treatments, but it was significantly lower under these treatments than under DAP and CK treatments, which were similar. Treatments had no influence on ALP activity, which was much higher than ACP activity (Fig. 5).

### Factors influencing soil cysts

**Fig. 6:**
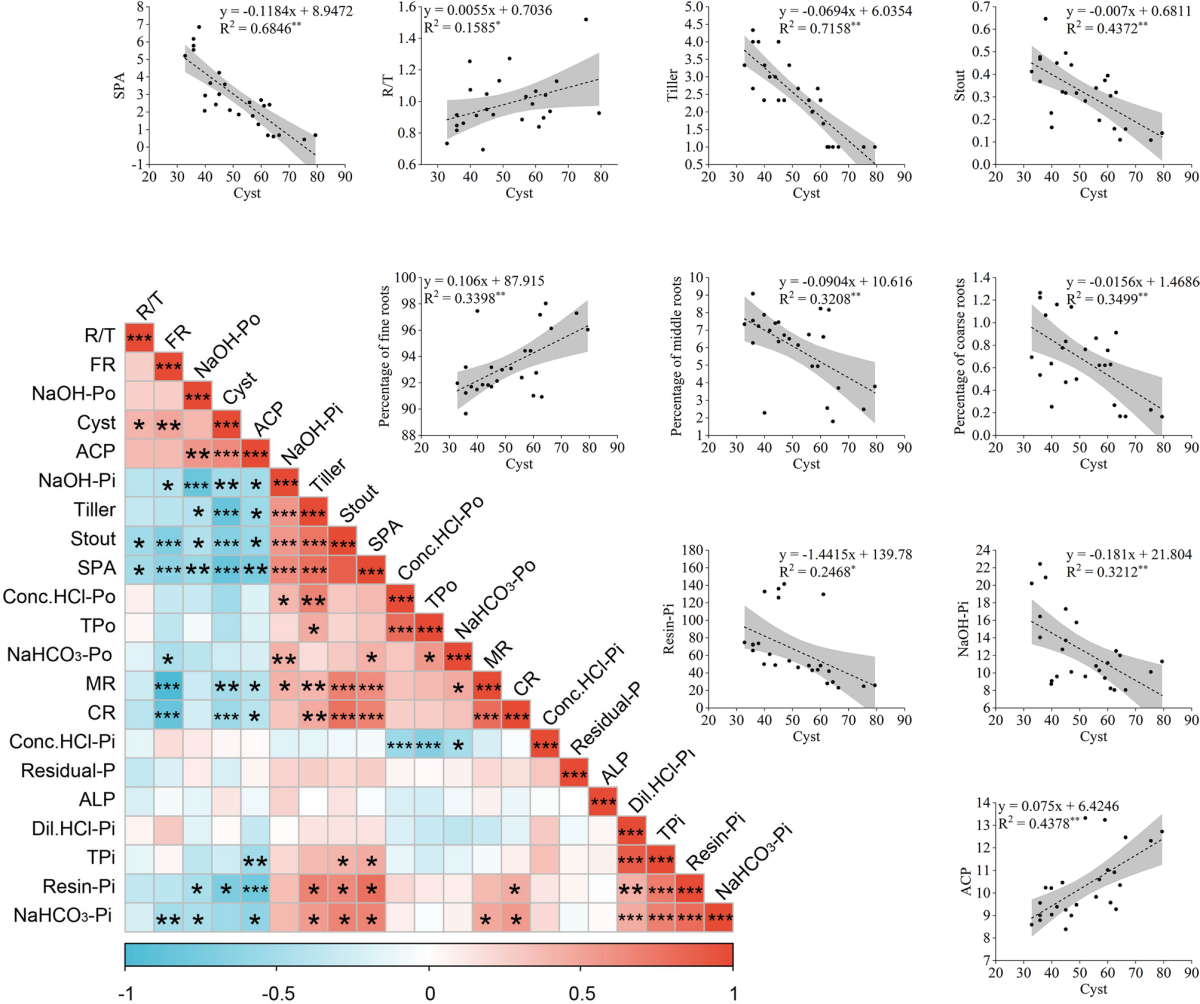
Interfactor correlations (heatmap) and linear regression of soil cysts with key factors. R/T, root-to-shoot ratio; Stout, seedling strength (ratio of shoot fresh weight to height); SPA, shoot P accumulation; FR, length ratio of fine roots (0–1 mm diameter); MR, length ratio of medium roots (1–3 mm diameter); CR, length ratio of coarse roots (> 3 mm diameter); TPo, total organic P; TPi, total inorganic P; ACP, acid phosphatase; ALP, alkaline phosphatase. Color gradient indicates correlation strength (red: positive; blue: negative). * represents *p* < 0.05, ** represents *p* < 0.01, ***represents *p* < 0.001

Correlation analysis was conducted to determine the relationships between environmental factors and CCN (Fig. 6). The correlation heatmap revealed seven factors exhibiting significant negative correlations with soil cysts (*p* < 0.05): shoot P accumulation (SPA), tiller number, stout, MR length proportion, CR length proportion, and Resin-Pi/NaOH-Pi concentrations (Table S2 and 3). Conversely, three factors exhibited significant positive correlations with soil cysts (*p* < 0.05): R/T ratio, FR length proportion, and ACP activity. Notably, all these key factors were demonstrated significant linear relationships with soil cysts. Moreover, concentrations of soil P fractions (Resin-Pi, NaHCO_3_-Pi/Po, NaOH-Pi, total Pi), root morphology (MR/CR length proportions), and tiller number all showed significant positive correlations with SPA (*p* < 0.05).

**Fig. 7:**
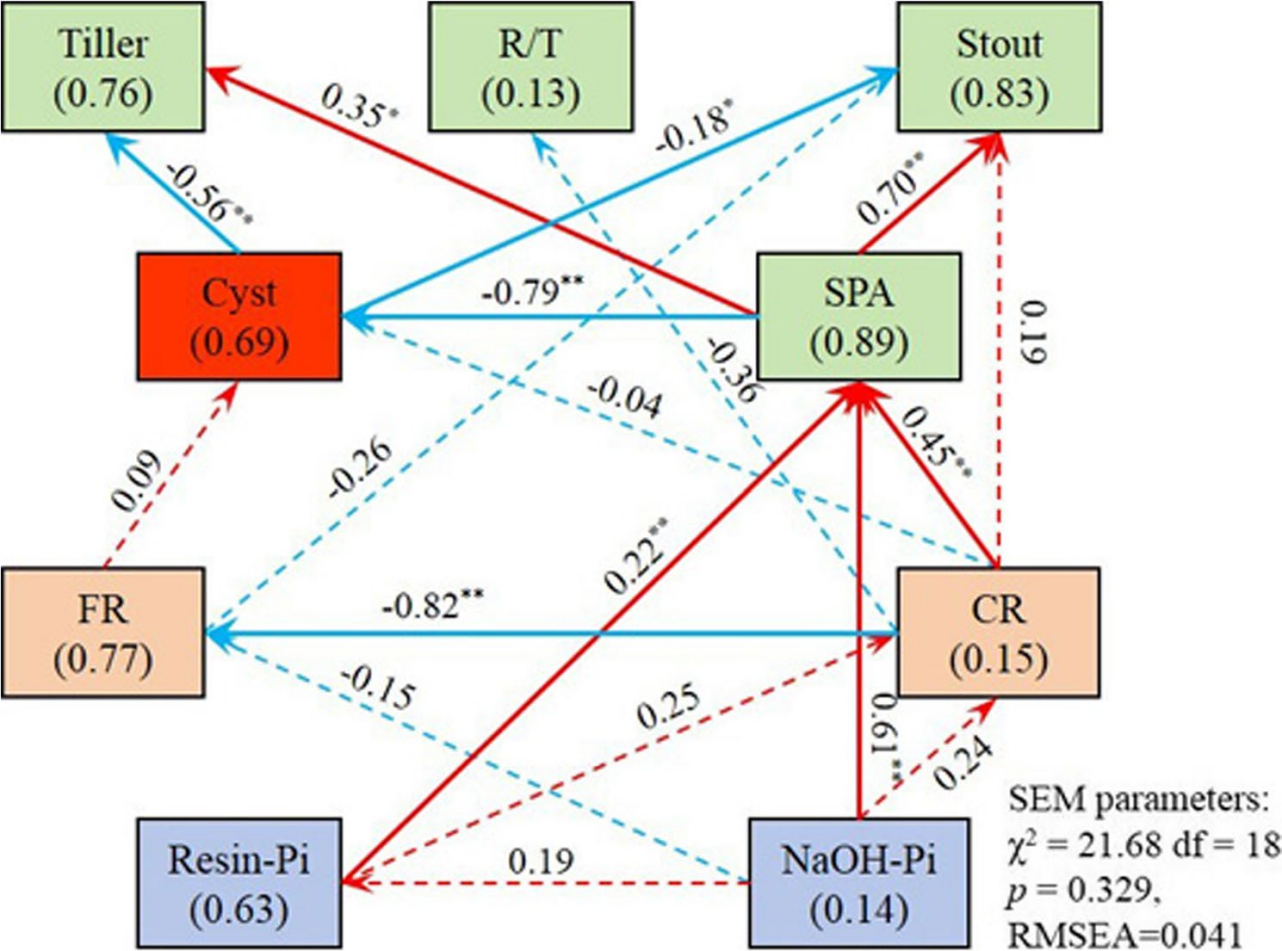
Structural equation modeling analysis of the effects of wheat biological traits and soil P fractions on CCN incidence. R/T, root-to-top ratio; Stout, seedling strength (ratio of shoot fresh weight to height); Cyst, cyst numbers; SPA, shoot P accumulation; FR, length proportion of fine roots (0–1 mm diameter); CR, length proportion of coarse roots (> 3 mm diameter). Red and blue arrows represent positive and negative relationships, respectively. Solid and dashed lines represent significant and non-significant pathways, respectively. The numbers in brackets in the squares are the amount of variation interpreted by variables, and the numbers on the arrows are the path coefficients. Sample size = 25. * represents *p* < 0.05, ** represents *p* < 0.01

Furthermore, a SEM was constructed to determine the effects of wheat biological traits and rhizosphere soil P fractions on the occurrence of CCN, and the results are shown in Fig. 7. The SEM analysis revealed that SPA suppressed number of cyst (*r* = −0.79**), while higher cyst counts in turn reduced tiller numbers (*r* = −0.56**) and stout of wheat plants (*r* = −0.18*). Therefore, the SPA exhibited a promotion effect on the tiller number (*r* = 0.35*) and stout (*r* = 0.7**). In addition, the SPA was promoted by Resin-Pi (*r* = 0.22**), NaOH-Pi (*r* = 0.14**), and CR (*r* = 0.45**).

## Discussion

P nutrition fundamentally shapes plant-pathogen dynamics by simultaneously modulating belowground ecological interactions and activating host defense mechanisms [14, 17]. Studies showed increases in soil P content with phosphate fertilizer application can lead to changes in the structure of the soil nematode community and inhibit the relative abundance of the PPN community [36, 37]. The soybean cyst nematode, *Heterodera glycines*, has shown a negative correlation with soil available P content in soybean fields [36]. Similarly, a negative correlation was found between soil available P regulated by fertilization and the occurrence of CCN (*Heterodera avenae*), suggesting a key role for soil P nutrition in inhibiting CCN [2]. However, the inhibition of CCN by different phosphate fertilizers was not the same. The application of MAP or DAP can reduce the number of cysts, with MAP demonstrating stronger inhibitory effect than DAP, consist with our experimental findings [19]. In this study, MAP and SSP showed comparable suppression effects on CCN, outperforming DAP. Notably, APP exhibited the highest inhibitory effect among all tested phosphate fertilizers. This distinct inhibition may be due to the amount of P absorbed by wheat under different phosphate fertilizer types, as phosphate can induce resistance to disease in plant [38]. Improving plant P nutrition can affect the response pathways evoked by PPN [38, 39]. Plant immune systems are directly linked to P nutrition [40]. P levels can serve as a signal in plants, directly impacting the immune response through phytohormone synthesis. Specific phytohormones, such as salicylic acid and jasmonic acid, mediate the biosynthesis of secondary metabolites involved in plant defense [41]. Phosphate fertilizer has been suggested to strengthen wheat immune system, enhancing CCN resistance with efficacy varying by fertilizer type [19, 40].

The application of phosphate fertilizers not only reduces the number of cysts, but also changes the concentration of soil P fractions [23, 30]. Yuan et al. [42] found that the concentration of NaHCO_3_-Pi under APP application was 2.4-fold higher than that under MAP. The superior performance of APP in supplying plant-available P, evidenced by its higher Resin-Pi concentrations relative to MAP and DAP, can be attributed to its distinct chemical behavior in highly alkaline soils [43]. Specifically, the polymeric form of APP exhibits weak binding affinity with calcium carbonate under alkaline conditions, thereby enhancing its effectiveness in increasing active P pools [44]. Nevertheless, whether similar outcomes occur in neutral or acidic soils remains uncertain, as soil pH exerts a critical influence on P solubility and precipitation kinetics. Overall, phosphate fertilization mainly enhances active P fractions, such as Resin-Pi, NaHCO_3_-Pi, and NaHCO_3_-Po [43]. Importantly, soil active P concentrations have been shown to be significantly and negatively correlated with soil-borne disease incidence [14]. For instance, Qiu et al. [2] reported that the fertilization-induced increases in soil active P were negatively associated with the incidence of CCN in wheat. Consistently, the present study revealed that correlations among Resin-Pi, SPA, and cyst numbers suggest a P-mediated suppression of CCN infection, likely through phosphorus-strengthened plant defense pathways [40]. Furthermore, the strong negative correlation between NaOH-Pi and cyst numbers implies that not only active but also moderately active P fractions may contribute to CCN suppression, although the underlying mechanisms require further exploration.

Beyond their impact on soil P fractions, phosphate fertilizers could potentially mediate soil-borne diseases through P-regulated changes in root morphology [45, 46]. Generally, P deficiency induces plants to form a larger root/top ratio, and promoting root branches helps plants absorb more P from soil, which is a response strategy for plant adaptation to P deficiency stress [46, 47]. The application of phosphate fertilizer mitigates P-deficiency stress and enables recovery of root morphology to non-stressed levels, with efficacy varying among fertilizer types [48]. Peng et al. [49] found that SSP application significantly decreased total root length of rice compared with DAP. When treated with APP, root diameter of maize was much greater than treated with DAP [48]. The observed divergence in root morphology traits under different phosphate fertilizers suggests form-specific modulation of root development [50]. Precisely, PPN infestation is affected by changing the root morphology of the host [51, 52]. Studies have shown that fertilization inhibits the abundance of PPN by reducing the volume of root, which is the primary food source of plant-feeding nematodes [51]. Specifically, the diameter of the roots may be one of the key factors between PPN and roots. In the case of the same volume, a smaller root diameter would be accompanied by an increase in root length, contact area between the PPN and the root system, number of feeding sites, and number of nematode infections [53]. Although the infected root diameter range was speculated to be 1–10 mm for *Heterodera* spp., *H. avenea* Woll may commonly attack small-diameter roots [53]. However, this requires more experimental data for support. The present study revealed that P fertilization-induced increases in coarse root length proportion (> 3 mm diameter) in wheat, consistent with known P effects on root architecture, showed a significant negative correlation with soil cysts [54]. Therefore, it would make sense that P fertilized, well P fed wheat plants have coarser root systems offering less opportunity to CCN, resulting in healthier and larger plants with high SPA.

In soil ecosystems, the transformation of inorganic and organic P is mainly driven by phosphatases, particularly ACP and ALP [23]. This study found that ACP activity was significantly lower than ALP activity under phosphate fertilizer applications, likely due to the alkaline calcareous soil used [55]. In addition, soil active P increases to meet the nutrient requirements of plants in SSP, MAP, and APP treatments, leading to a decrease in soil ACP activity [42]. ACP activity is generally correlated with soil organic P utilization [56, 57]. When inorganic P cannot meet the requirements for plant growth, plants secrete phosphatases to promote the mineralization of organic P into inorganic form, thus facilitating the conversion between soil P pools [12]. This study showed that the soil ACP activity was significantly positively correlated with NaOH-Po but negatively correlated with Resin-Pi, NaHCO_3_-Pi, and NaOH-Pi. It has been suggested that ACP mainly functions in the mineralization of NaOH-Po to form plant-available inorganic P, thereby enhancing plant P nutrition for disease defense [14]. Moreover, an increase in soil ACP activity under P deficiency can promote plant P uptake, sometimes exceeding the P requirements for plants [58]. Since ACP is both plant- and microbial-derived, its activity variation under different phosphate fertilization treatments may reflect responses from both groups [30]. However, the role of microbes and their specific mechanisms in CCN suppression under phosphate fertilization conditions require further investigation.

## Conclusions

All the phosphate fertilizers tested showed a significant inhibitory effect on cyst numbers. It is suggested that the concentration of Resin-Pi increased by phosphate fertilizer additions, enhancing shoot P accumulation and inhibiting the occurrence of wheat CCN. In addition, well P fed wheat plants have coarser root systems offering less opportunity to CCN, resulting in healthier and larger plants. Notably, NaOH-Pi concentration in rhizosphere soil increased significantly in APP treatment correlated with the highest shoot P accumulation and biomass, indicating that P nutrition provided by APP is more easily absorbed by wheat than the other tested fertilizers. However, the underlying mechanism by which moderately active P mediates CCN occurrence still needs further exploration. Therefore, as a superior P source, APP not only promotes wheat growth by enhancing P uptake, but also increases the resistance of wheat against CCN.

## Supplementary Information

Supplementary Material 1.

Supplementary Material 2.

## Data Availability

The datasets used and/or analyzed during the current study are available from the corresponding author on reasonable request.

## Abbreviations

CCN: Cereal cyst nematode
PPN: Plant parasitic nematode
P: Phosphorus
SSP: Single superphosphate
MAP: Monoammonium phosphate
DAP: Diammonium phosphate
APP: Ammonium polyphosphate
ACP: Acid phosphatase
ALP: Alkaline phosphatase
SEM: Structural equation model
FR: Fine root
MR: Medium root
CR: Coarse root
