# Supplementary material for: Rhizosphere phosphorus fractions controlled through P fertilization influence wheat infection by Heterodera avenae
Source: BMC Plant Biol. 2025 Oct 3;25:1299. doi: 10.1186/s12870-025-07399-5 (PMC12495683; doi:10.1186/s12870-025-07399-5)
Supplement: Supplementary file 1 — Supplementary Material 1. [file 12870_2025_7399_MOESM1_ESM.docx]

Article title: **Rhizosphere phosphorus fractions controlled through P fertilization influence wheat infection by *Heterodera avenae***

Journal name: *BMC Plant Biology*

Author names: Mengli Zhao^1,†^, Pengfei Wang^1,†^, Xiuli Dong^1^, Siyao Huang^1^, Cihong Wang^1^, Jun Yuan^2^, Wei Qiu^1,*^, Junhui Chen^1^

Affiliation:

^1^College of Environment and Resources, College of Carbon Neutral, Zhejiang Agriculture and Forestry University, Hangzhou, Zhejiang 311300, China

^2^Key Lab of Organic‑Based Fertilizers of China, Jiangsu Provincial Key Lab for Solid Organic Waste Utilization, Jiangsu Collaborative Innovation Center of Solid Organic Wastes, Educational Ministry Engineering Center of Resource‑Saving Fertilizers, Nanjing Agricultural University, Nanjing, Jiangsu 210095, China.

*Corresponding author. Email: qiuwei@zafu.edu.cn.

^†^These authors have contributed equally to this work.


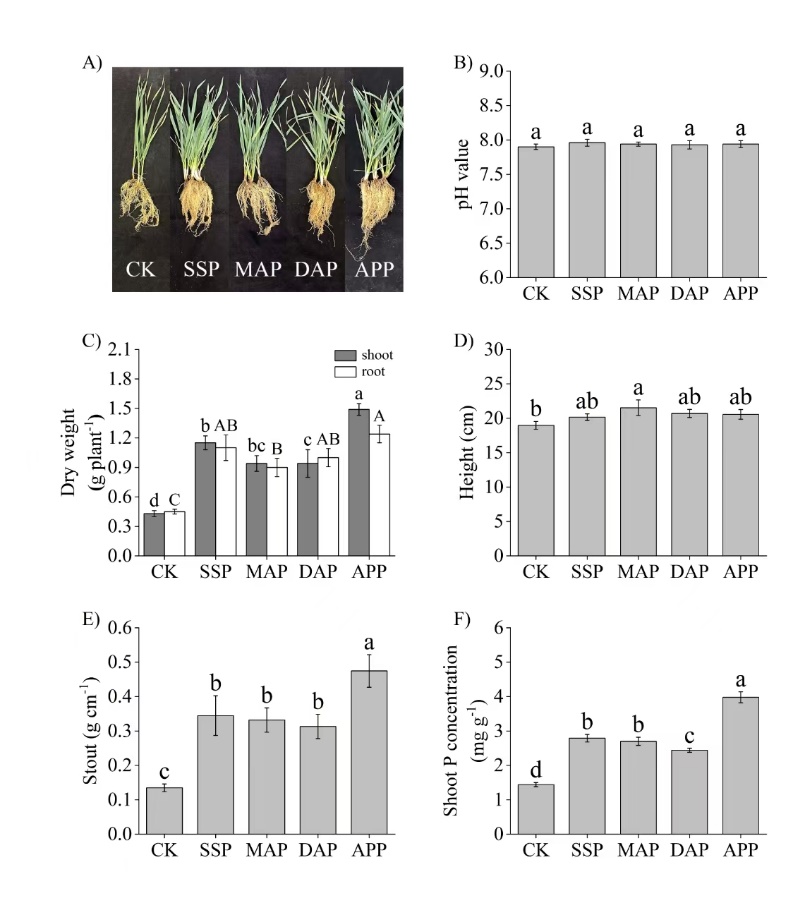


**Fig. S1** Effects of phosphate fertilizers on the (A) wheat growth, (B) soil pH value, (C) dry weight, (D) height, (E) stout (seedling strength, ratio of shoot fresh weight to height), and (F) shoot P concentration. SSP, single superphosphate; MAP, monoammonium phosphate; DAP, diammonium phosphate; APP, ammonium polyphosphate; CK, without phosphate fertilizer. Each value is the mean ± SE of four replicates. Different letters show statistically significant differences among treatments (*p* < 0.05).
